# Inhibition of GADD34, the Stress-Inducible Regulatory Subunit of the Endoplasmic Reticulum Stress Response, Does Not Enhance Functional Recovery after Spinal Cord Injury

**DOI:** 10.1371/journal.pone.0109703

**Published:** 2014-11-11

**Authors:** Sujata Saraswat Ohri, Ashley Mullins, Michal Hetman, Scott R. Whittemore

**Affiliations:** 1 Kentucky Spinal Cord Injury Research Center, University of Louisville, Louisville, Kentucky, United States of America; 2 Department of Neurological Surgery, University of Louisville, Louisville, Kentucky, United States of America; 3 Department of Pharmacology & Toxicology, University of Louisville, Louisville, Kentucky, United States of America; 4 Department of Anatomical Sciences & Neurobiology, University of Louisville, Louisville, Kentucky, United States of America; Massachusetts General Hospital/Harvard Medical School, United States of America

## Abstract

Activation of the endoplasmic reticulum stress response (ERSR) is a hallmark of various pathological diseases and/or traumatic injuries. Restoration of ER homeostasis can contribute to improvement in the functional outcome of these diseases. Using genetic and pharmacological inhibition of the PERK-CHOP arm of the ERSR, we recently demonstrated improvements in hindlimb locomotion after spinal cord injury (SCI) and implicated oligodendrocyte survival as a potential mechanism. Here, we investigated the contribution of stress-inducible PPP1R15A/GADD34, an ERSR signaling effector downstream of CHOP that dephosphorylates eIF2α, in the pathogenesis of SCI. We show that although genetic ablation of GADD34 protects oligodendrocyte precursor cells (OPCs) against ER stress-mediated cell death *in vitro* and results in differential ERSR attenuation *in vivo* after SCI, there is no improvement in hindlimb locomotor function. Guanabenz, a FDA approved antihypertensive drug, was recently shown to reduce the burden of misfolded proteins in the ER by directly targeting GADD34. Guanabenz protected OPCs from ER stress-mediated cell death *in vitro* and attenuated the ERSR *in vivo* after SCI. However, guanabenz administration failed to rescue the locomotor deficits after SCI. These data suggest that deletion of GADD34 alone is not sufficient to improve functional recovery after SCI.

## Introduction

Spinal cord injury (SCI) is a complex multifactorial pathological condition and strategies to discover new effective therapies remain challenging. Therapeutic intervention has been difficult due to the management of two complex pathological phases following SCI: primary mechanical injury of which some level of cell death and axotomy occurs depending on the severity of the injury, and secondary damage initiated by the primary trauma. Secondary injury mechanisms that include inflammation, hypoxia, excitotoxicity, ischemia and demyelination exacerbate the primary damage [1]. Consequently, rapid cellular necrosis occurs at the injury epicenter with onset of apoptosis that spreads out into the lesion penumbra [2] and contributes to secondary cell death [1], [2]. Therapeutic interventions which abrogate secondary cell death mechanisms during the acute phase of SCI are essential for limiting the spread of secondary injury phase and functional deficits after SCI.

While preclinical studies have identified a number of potential neuroprotective agents [3], [4], none of these have shown marked therapeutic efficacy in human SCI trials [5], [6]. One likely explanation is that multiple pathophysiological mechanisms are being activated simultaneously and current single drug therapies only affect a subset of these. What is hypothetically warranted for optimal therapeutic treatment would be pharmacological therapy that globally targets multiple neuropathological mechanisms in all cell types affected. One such potential target that has proven effective in the acute treatment of SCI is the endoplasmic reticulum (ER) stress response (ERSR).

The ERSR is an evolutionary conserved cellular mechanism that is activated in response to insults that disrupt ER homeostasis [7], [8]. It is mediated by three distinct pathways: the protein RNA (PKR)-like kinase (PERK), inositol-requiring protein-1α (IRE-1α) and activating transcription factor-6 (ATF6). Activated PERK phosphorylates the α-subunit of elongation initiation factor 2α (eIF2α) which then results in global inhibition of protein synthesis. Moreover, phosphorylation of eIF2α allows the translation of mRNAs containing short open reading frames in their 5′-untranslated regions, such as activating transcription factor 4 (ATF4). Activated IRE-1α splices the X-box-binding protein 1 (XBP1) mRNA [9] and activates ERSR-specific transcription. Proteolytic processing of ATF6 [10] at the golgi complex also results in distinct expression of ERSR-specific genes. The main objective of the ERSR is cytoprotection by restoring ER homeostasis. However, the ERSR may also activate apoptosis if ER function cannot be timely restored, an event triggered by increased C/EBP homologous protein (CHOP) expression [8].

Dephosphorylation of eIF2α is required to restore protein synthesis after the stress induced attenuation of translation, thereby, terminating stress signaling [11]. Mammalian cells have two eIF2α holophosphatases, each one composed of one PP1 catalytic subunit and either PPP1R15A (growth arrest and DNA damage protein 34 - GADD34) or PPP1R15B (CReP). GADD34 is transcriptionally induced by stress, downstream of ATF4 and CHOP, and its translation escapes the general attenuation of protein synthesis resulting from eIF2α phosphorylation [12]. Thus, GADD34/PPP1R15A is one of the key effectors of negative feedback loop that is induced by stress and subsequently tries to restore homeostasis. Recent studies show that expression of GADD34 sensitizes cells to apoptosis [12], [13], [14]. Studies using GADD34^-/-^ mice showed diminished oligodendrocyte loss and hypomyelination in experimental autoimmune encephalomyelitis [15] and improved myelination of Schwann cells in model of Charcot-Marie-Tooth IB neuropathy [16].

Independent studies demonstrated the involvement of the ERSR after a moderate contusive SCI in rats [17] and mice [18] or after hemisection SCI in mice [19]. Partial restoration of ER homeostasis using genetic [18], viral [19], or pharmacological [20] interventions showed significant improvement of hindlimb locomotion in these rodent models. Interestingly, our previous studies demonstrated significant upregulation of GADD34 after both moderate and severe SCI [18], [21]. Guanabenz, an α2-adrenergic receptor agonist, FDA-approved for the treatment of hypertension, was recently shown to enhance the PERK pathway by selectively inhibiting GADD34-mediated dephosphorylation of eIF2α [22]. Given its FDA-approved status, this study was undertaken to study the therapeutic potential of guanabenz and GADD34 inhibition in modulating the ERSR after SCI.

## Materials and Methods

### Animals

Wild type C57Bl/6 female mice (6–8 weeks) were obtained from Harlan (Indianapolis, IN). GADD34^-/-^ mice on a 100% C57Bl/6 background were procured from MMRRC (Catalogue No – 30266, Chapel Hill, NC) and bred in-house. Procedures were performed according to the Public Health Service Policy on Humane Care and Use of Laboratory Animals, Guide for the Care and Use of Laboratory Animals (Institute of Laboratory Animal Resources, National Research Council, 1996) and with the approval of the University of Louisville Institutional Animal Care and Use Committee and the Institutional Biosafety Committee.

### Isolation of mouse oligodendrocyte precursor cells (mOPCs)

Mouse cortices were dissected from whole brains of wild type and GADD34^-/-^ postnatal day 5–7 pups [23]. Briefly, tissue was dissociated using the Neural Tissue Dissociation Kit (Miltenyi Biotec, Bergisch Gladbach, Germany) according to the manufacturer's instructions. OPC-A media was prepared by adding 2.1 g/L NaHCO_3_ (Sigma-Aldrich, St. Louis, MO) to DMEM-F12 without HEPES powder (Invitrogen, Carlsbad, CA) and supplemented with N2 supplement (1%), B27 supplement (2%), Penicillin/Streptomycin (1%, all from Invitrogen), BSA (0.01%, Sigma), 40 ng/ml FGF2 (Millipore, Billerica, MA), and 20 ng/ml PDGFα (Sigma). OPCs were enriched with O4 hybridoma using magnetic cell sorting (MACS) with rat anti-mouse IgM magnetic beads (10% in MACS Buffer). The average yield was 8 −10×10^6^ cells/brain with a viability of 85–95%. 9,000–15,000 cells/cm^2^ cells were seeded on a PDL/laminin-coated 10 cm tissue culture dish, and incubated at 37°C, 5% CO_2_ for maintenance.

### Tunicamycin treatment, MTT assay

Wild type or GADD34^-/-^-derived mOPCs were seeded in 96-well plates and treated with various concentrations of guanabenz in the presence of tunicamycin (0.01 or 0.025 µg/ml). OPC survival was assayed by measuring the conversion of tetrazolium, MTT (3-(4,5-dimethylthiazol-2-yl)-2,5-diphenyltetrazolium bromide; Sigma) to formazan at a wavelength of 570 nm.

### Western Blot Analyses

Protein lysates prepared from WT and GADD34^-/-^-derived mOPCs or 4 mm tissue isolated from sham and injury epicenter of contused spinal cords at 6 hour post-injury in protein lysis solution (20 mM Tris, pH-6.8, 137 mM NaCl, 25 mM B-glycerophosphate, 2 mM NaPPi, 2 mM EDTA, 1 mM Na3VO4, 1% Triton X-100, 10% glycerol, protease inhibitor, 0.5 mM DTT, 1 mM PMSF) were quantified using the BCA Kit (Pierce). Proteins were separated on SDS-PAGE gels and transferred to nitrocellulose membrane (Whatman, Schleicher & Schuell). Membranes were processed and probed with p-eIF2α (Cell Signalling, 1/1000 dilution), eIF2α (Biosource, 1/1000), ATF4 (Santa Cruz Biotechnology, 1/500) and GAPDH (Chemicon, 1/5000) as described previously [20].

### RNA Extraction, Reverse Transcriptase PCR

Total RNA was extracted from WT and GADD34^-/-^-derived mOPCs treated with tunicamycin and spinal cord tissue of sham, vehicle- and guanabenz-treated contused wild type (n = 4/group) mice from the injury epicenter (4 mm) using Trizol (Invitrogen, Carlsbad, CA) according to the manufacturer's instructions. The RNA was quantified by UV spectroscopy and RNA integrity was confirmed on an ethidium bromide stained formaldehyde agarose gel. cDNA was synthesized with 500 ng of total RNA using the High Capacity cDNA Synthesis Kit (Applied Biosystems, Foster City, CA) in a 20 µl reaction volume. As controls, mixtures containing all components except the reverse transcriptase enzyme were prepared and treated similarly. All cDNAs and control reactions were diluted 10x with water before using as a template for quantitative real time (qRT)-PCR.

### Quantitative PCR Analysis

qRT-PCR was performed using ABI 7900HT Real-time PCR instrument (Applied Biosystems). Briefly, diluted cDNAs were added to Taqman universal PCR master mix (Applied Biosystems) and run in triplicate. Target and reference gene PCR amplification was performed in separate tubes with Assay on Demand™ primers (Applied Biosystems) as follows: ATF4 (Mm00515324_m1), CHOP (Mm01135937_g1), Claudin 11 (Mm00500915_m1) GADD34 (Mm00492555_m1), GFAP (Mm00546086_m1), glutamine synthetase (GS: Mm00725701_s1), GRP78 (Mm01333323_g1), microtubule associated protein 2 (Mtap2: Mm00485230_m1), myelin basic protein (MBP: Mm00521980) neuron-specific enolase (NSE: Mm00468052_m1) Olig2 (Mm01210556_m1) and XBP1 (Mm00457359_m1). The RNA levels were quantified using the ΔΔCT method. Expression values obtained from triplicate runs of each cDNA sample were normalized to triplicate value for GAPDH (reference gene) from the same cDNA preparation. Transcript levels are expressed as fold changes compared with respective levels in sham controls.

### XBP1 Splicing

qRT-PCR with RT^2^ Real-Time SYBR Green Mix (SuperArray Bioscience Corporation) using spliced XBP1 (sense, gagtccgcagcaggtg; antisense, gtgtcagagtccatggga) [24] and GAPDH (sense, ccctcaagattgtcagcctgc; antisense, gtcctcagtgtagcccaggat) primers was performed. The ΔΔCT method was used for quantification.

### SCI and injections

Mice were anesthesized by an intraperitoneal (IP) injection of 0.4 mg/g body weight Avertin (2,2,2-tribromoethanol in 0.02 ml of 1.25% 2-methyl-2-butanol in saline, Sigma). Lacri-Lube ophthalmic ointment (Allergen, Irvine, CA) was used to prevent drying of eyes and gentamycin (50 mg/kg; Boehringer Ingelheim, St. Joseph, MO) was subcutaneously administered to reduce infection. A laminectomy was done at the T9 vertebrae and moderate contusion injuries (50 kdyn force/400–600 µm displacement) were performed using the IH impactor [25] (Infinite Horizons Inc., Lexington, KY) as described previously [26], [27]. Experimental controls included sham animals that received only the T9 laminectomy. Guanabenz (1 mg/kg; in PBS containing 10% FBS and 5% DMSO) or vehicle was administered by an IP injection immediately after surgery followed by IP injections for three consecutive days. Post surgery, animals were given 1 cc of sterile saline subcutaneously, 0.1 cc of gentamycin intramuscularly on the day of surgery and 3^rd^ and 5^th^ day post-surgery, and 0.1 cc bupronorphine subcutaneously on the day of surgery and for next 2 days. Animals were placed on a heating pad until full recovery from anesthesia. Postoperative care included manual expression of bladders twice a day for 7–10 days or until spontaneous voiding returned.

### Behavioral Assessment

Open field Basso Mouse Scale (BMS) locomotor analyses were performed prior to injury for each animal to determine the baseline scores and weekly following SCI for 6 weeks exactly as defined [28], 20. All raters were trained by Dr. Basso and colleagues at the Ohio State University and were blind to the animal groups.

### Statistical Analyses

For functional assessements after injury, a repeated measures analyses of variance (ANOVA) with fixed effects and Bonferroni post hoc t-test was performed to detect differences in BMS score and subscores between the sham and injury groups over the 6 week testing period. Statistical analysis of qRT-PCR data was performed using independent t-test for means with equal or unequal variances or repeated measures ANOVA (one- way or two-way analysis of variance) followed by post-hoc Tukey HSD test. For all other analyses, independent t-tests for means assuming equal variance were performed.

## Results

### GADD34^-/-^ mOPCs show a delayed decrease in survival after ER stress

**Figure 1:**
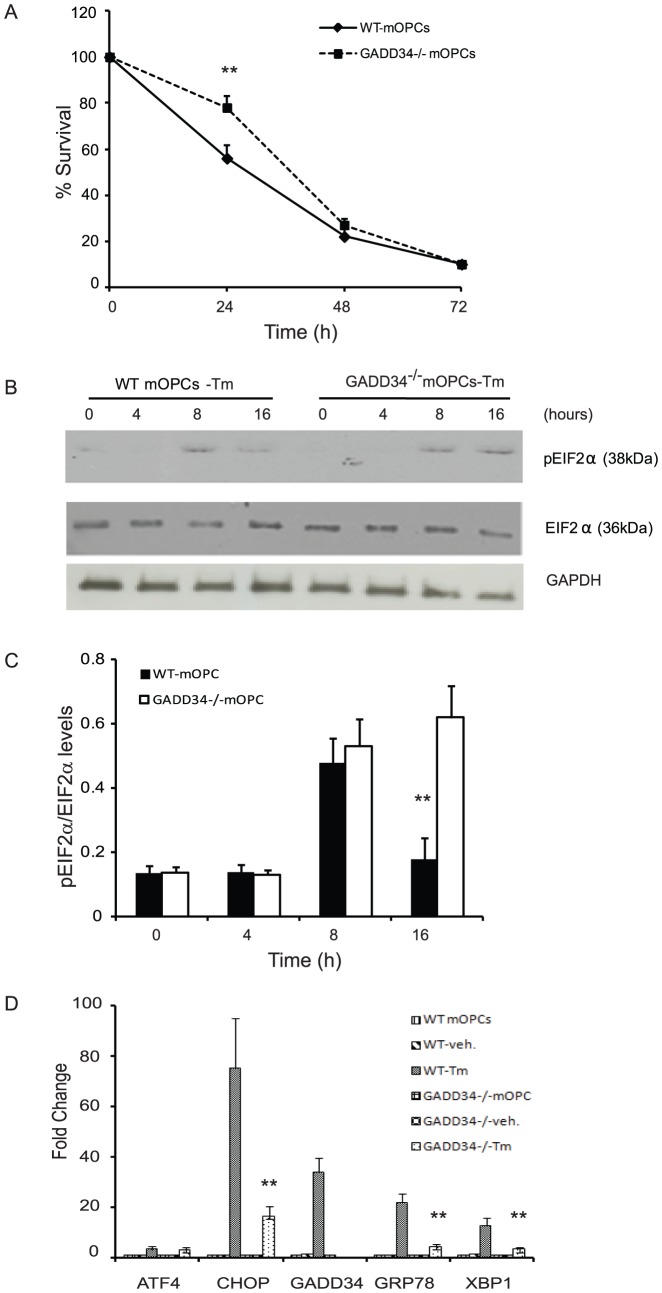
GADD34^-/-^ mOPCS show enhanced survival in response to ER stress. (A) Quantification using MTT assay show increased survival of GADD34^-/-^ mOPCs exposed to tunicamycin (Tm; 0.01 µg/ml) at only 24 hours. (B,C) Western blot shows sustained translational repression in GADD34^-/-^ mOPCs in contrast to WT mOPCs. (D) RT-PCR data shows attenuation in the ERSR in GADD34^-/-^ mOPCs exposed to Tm for 16 hours. Data (A,C,D) are the mean ± SD (n = 4, ** p<0.01).

The PPP1R15A/GADD34 complex, a regulatory subunit of protein phosphatase 1, selectively disrupts the stress-induced phosphorylation of eIF2α [29], [22]. Our previous studies that showed enhanced functional recovery post-SCI in CHOP^-/-^ mice and suggested oligodendrocyte sparing as a potential mechanism also showed immediate increase in GADD34 transcript levels after moderate SCI [18]. To explore the specific role of GADD34 in OPCs, we first compared the ability of wild type and GADD34^-/-^ mOPCs to survive exposure to ER stress induced by tunicamycin. GADD34^-/-^ mOPCs showed a significant increase in survival only at 24 hours post-treatment (Fig 1A) suggesting acute oligodendrocyte protection. We examined the activation of ERSR in GADD34^-/-^ mOPCs exposed to ER stress. In WT mOPCs, tunicamycin treatment resulted in profound but transient increase of eIF2α phosphorylation. In contrast, GADD34^-/-^ mOPCs showed persistent phosphorylation (Fig 1B,C) and correlated with a decrease in the induction of CHOP, GRP78 and XBP1 transcript levels in response to ER stress (Fig 1D). Together, these data show that GADD34 is one of the key effectors that affects both the translational de-repression and stress-induced gene expression in mOPCs, consistent with earlier studies performed in fibroblasts [29], [30], [31].

### Deletion of GADD34 results in differential ERSR attenuation after SCI

**Figure 2:**
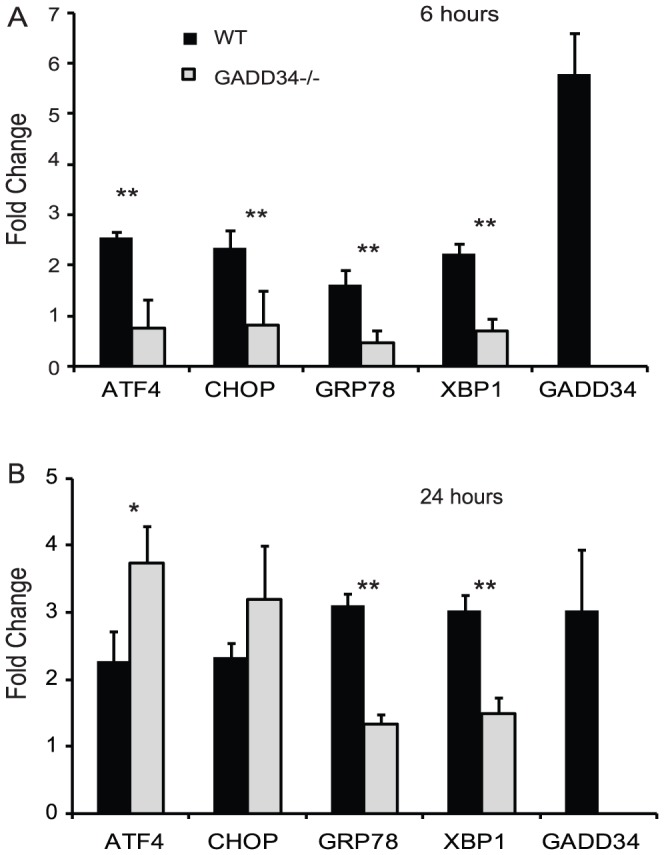
Deletion of GADD34 results in attenuation of the ERSR after SCI. Total RNA was extracted from injury epicenter of WT and GADD34^-/-^ mice at (A) 6 hours and (B) 24 hours-post SCI. Transcript level (normalized to GAPDH) is expressed as fold changes compared with levels in sham controls. Data (A,B) are the mean ± SD (n = 4, ** p<0.01).

To determine the contribution of GADD34 to SCI pathogenesis, basal levels of ERSR effectors in GADD34^-/-^ mice were compared with wild type mice. The average Ct values of 3.96 vs 3.872 for ATF4, 8.77 vs 8.44 for CHOP, 4.98 vs 5.12 for GRP78 and 6.077 vs 5.78 for XBP1 in GADD34^-/-^ and WT mice respectively, indicated identical basal ERSR in both groups. However, six hours post-SCI, GADD34^-/-^ mice demonstrated a significant reduction in the expression of ATF4, CHOP, GRP78 and XBP1 transcript levels (Fig 2A) indicating an overall attenuation of the ERSR in response to SCI. As the increased survival of GADD34^-/-^ mOPCs was transient, (Fig 1A), we also examined the ERSR at 24 hour post-SCI. The decrease in ATF4 and CHOP transcript levels observed at 6 hours was completely abolished at 24 hours post-SCI, while GRP78 and XBP1 transcript levels remained attenuated (Fig 2B).

**Figure 3:**
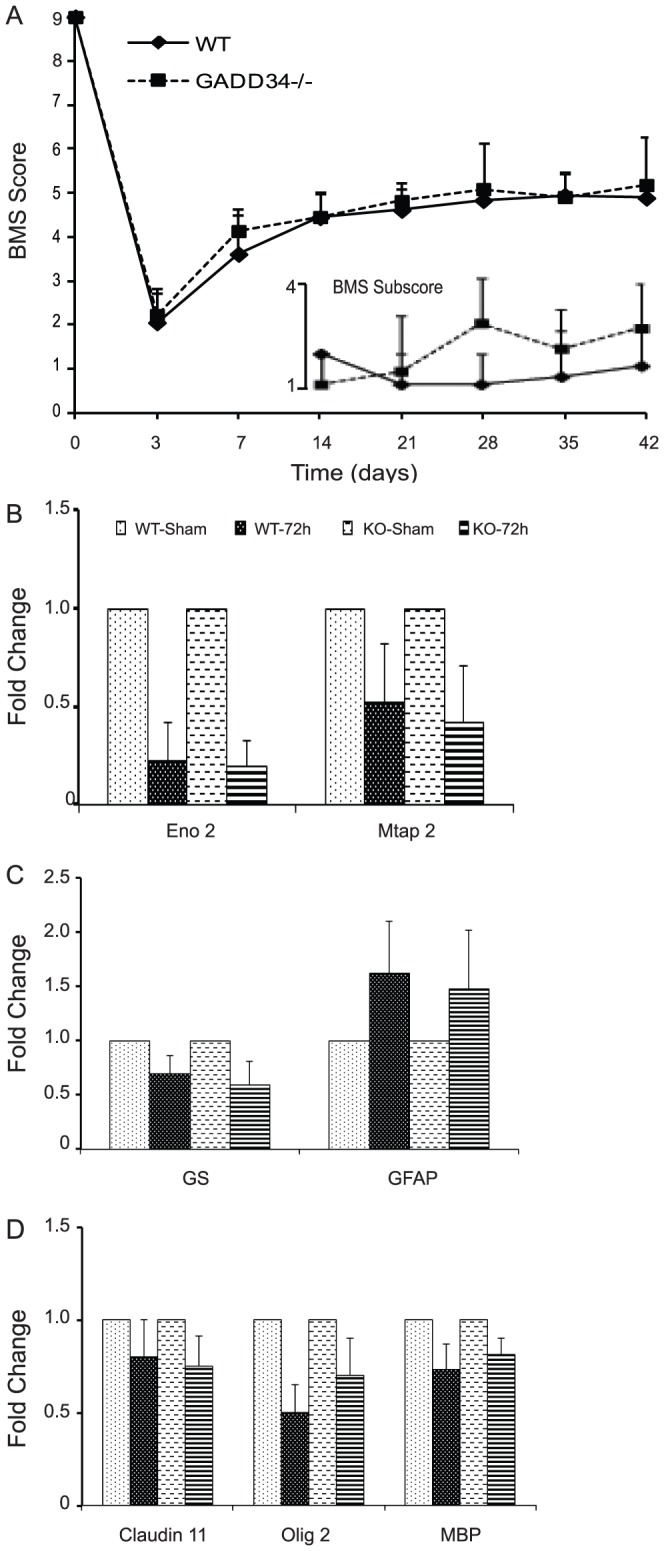
Locomotor assessment in GADD34^-/-^ mice after SCI. (A) BMS locomotor analyses performed weekly following moderate SCI did not reveal any functional recovery in GADD34^-/-^ mice (solid squares) compared to WT mice (solid diamonds). Analysis of BMS subscore post-SCI showed no significant differences in stepping characteristics between the two groups (inset). There is no significant difference in the neuron- (B), astrocyte-(C) and oligodendrocyte-specific (D) transcript levels as indicated between WT and GADD34^-/-^ mice 72 hours post-SCI. Data (A,B) are the mean ± SD (n = 9).

We evaluated the role of GADD34 in functional recovery post-SCI. Comparison of the BMS scores between moderately contused WT (n = 9) and GADD34^-/-^ (n = 11) mice revealed no differences. The average BMS score for WT and GADD34^-/-^ animals was 3.61±0.89 and 4.14±0.5 at week 1 and 4.89±0.42 and 5.18±1.08 at week 6, respectively (Fig 3A). Analysis of the BMS subscore also did not show any improvement in the stepping characteristics between WT and GADD34^-/-^ mice (Fig 3A, inset). We next determined the effect of GADD34 deletion on the survival of CNS-resident cells acutely after SCI. There were no significant differences in the neuron- (Fig 3B; NSE and Map2a,b), astrocyte- (Fig 3C; glutamine synthetase and GFAP) and oligodendrocyte-specific (Fig 3D; Claudin 11, Olig2, MBP) transcript levels in GADD34^-/-^ mice compared to WT mice at 72 hours post-SCI and is in contrast to our earlier study done in CHOP^-/-^ mice [18].

**Figure 4:**
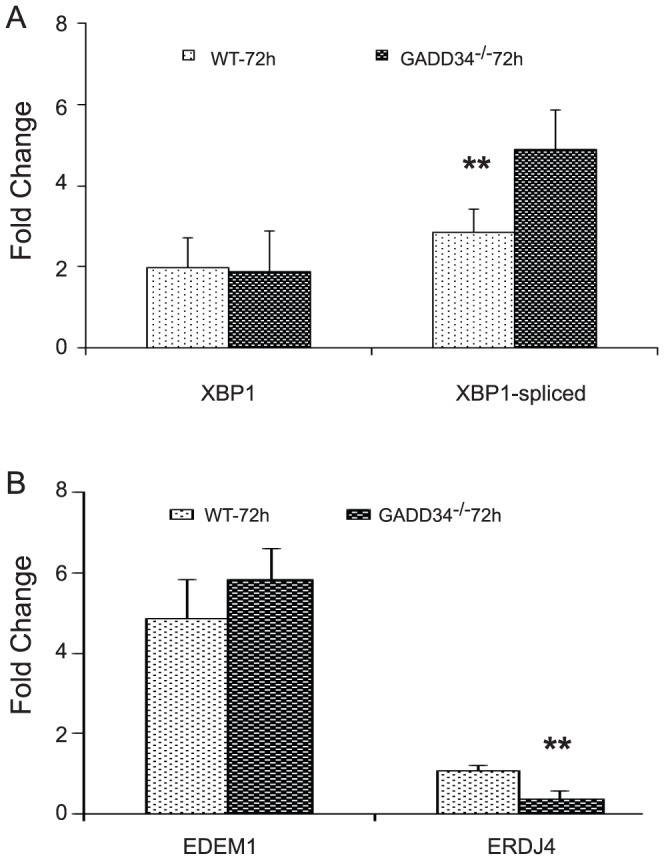
Expression of spliced XBP1 and its downstream target genes. qRT-PCR data shows significant differences in transcript levels of spliced XBP1 (A) and ERDJ4 (B) in GADD34^-/-^ mice compared to WT mice at 72 hours post-SCI.

To potentially explain the lack of functional recovery, levels of spliced XBP1 and its downstream target genes were evaluated in WT and GADD34^-/-^ mice 72 hours post-SCI to account for possible compensatory changes. A ∼2 fold upregulation of XBP1 mRNA in WT mice is consistent with our previous study [18] and is similar to XBP1 levels in GADD34 ^-/-^ mice. Interestingly, transcript level of spliced XBP1 is significantly higher in GADD34^-/-^ mice compared to wild type mice (Fig 4A). In addition, of the two target genes of XBP1 tested (EDEM1 and ERDJ4) [32], ERDJ4 mRNA level is significantly reduced in GADD34^-/-^ mice (Fig 4B).

### Guanabenz modestly promotes the survival of mouse OPCs and delays translational recovery

**Figure 5:**
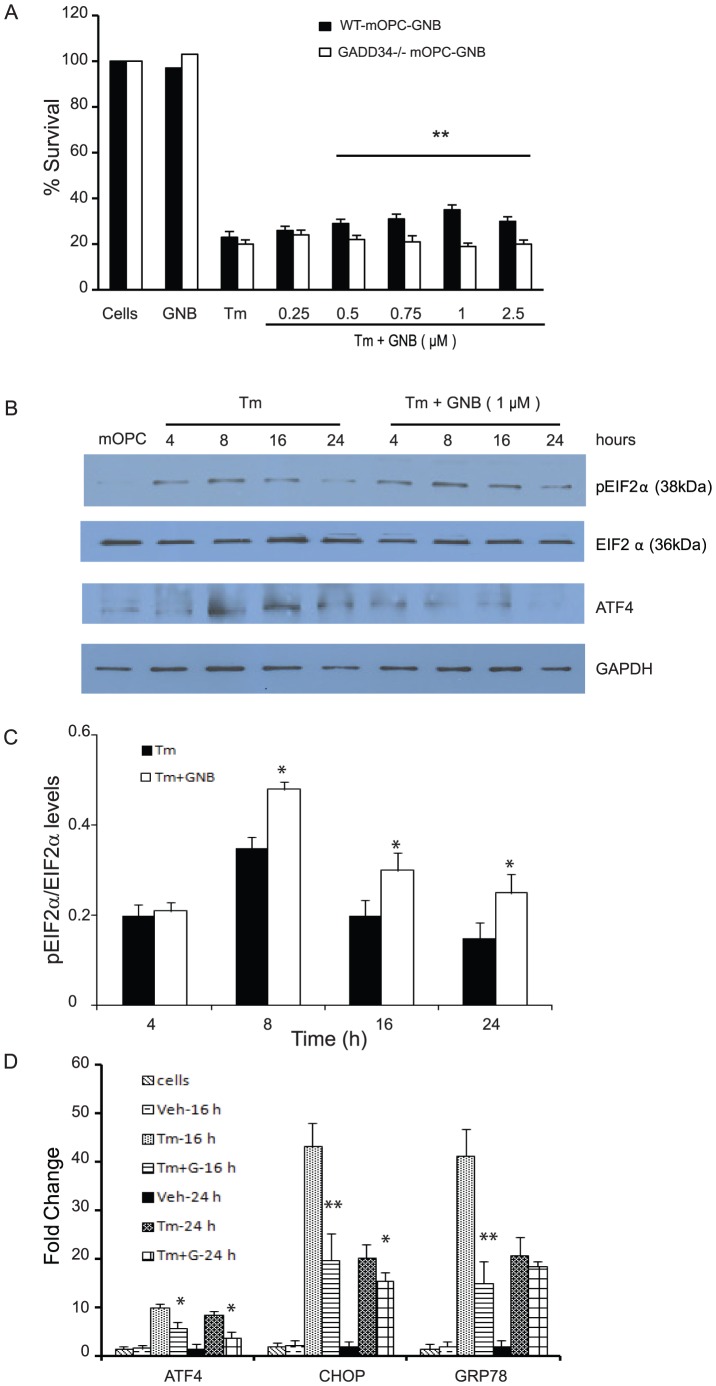
Effect of Guanabenz on WT and GADD34^-/-^ mOPCs. (A) Guanabenz modestly increases survival of WT but not GADD34^-/-^ mOPCs exposed to Tm (0.025 µg/ml) at 24 hours post-treatment assessed by MTT assay. (B,C) Western blot shows that guanabenz delays translational recovery in WT mOPCs treated with Tm. (D) Guanabenz leads to reduction in ATF4, CHOP and GRP78 transcript levels at 16 and 24 hours post-treatment. Data are the mean ± SD (n = 4; *p<0.05, ** p<0.01).

Guanabenz, an FDA approved antihypertensive drug, was recently identified to display anti-prion activity [33]. It also successfully corrected proteostasis defects by selectively targeting GADD34 and disrupting the stress-induced dephosphorylation of eIF2α in Hela cells [22]. In wild type mOPCs, guanabenz led to a maximum of 10% enhanced survival in response to cytotoxic ER stress induced by tunicamycin in a dose-dependent manner (Fig 5A). In contrast, GADD34^-/-^ mOPCs did not show any improvement in survival (Fig 5A) indicating that the cytoprotective activity of guanabenz in ER-stressed cells results from its inhibition of GADD34.

To delineate the mechanism(s) involved, the direct effects of guanabenz on stressed mOPCs were determined. Translational attenuation, evidenced by increased eIF2α phosphorylation, 4 hours after addition of tunicamycin, was not changed by guanabenz. However, translational recovery in OPCs treated with tunicamycin was markedly delayed in the presence of guanabenz as determined by evident levels of p-eIF2α at 24 hours (Fig 5B,C). In addition, guanabenz significantly attenuated tunicamycin-induced expression of ATF4, CHOP and GRP78 levels (Fig 5B,D). These data indicate that guanabenz modestly promoted the survival of mOPCs by targeting the PERK-eIF2α pathway of ERSR, consistent with previous data in Hela cells [22].

### Guanabenz enhances eIF2α phosphorylation after SCI

**Figure 6:**
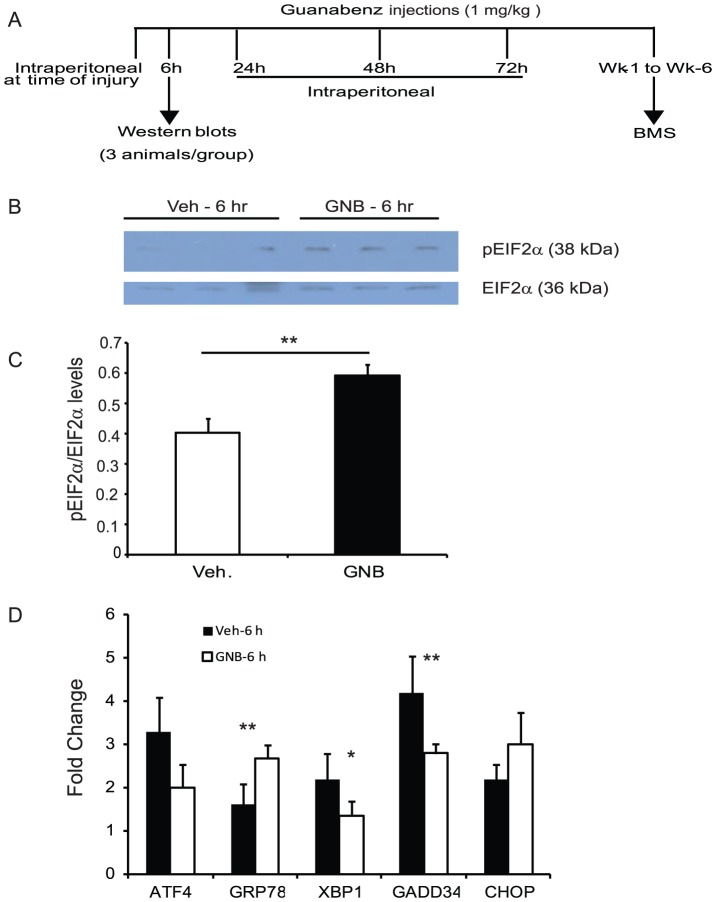
*In vivo* administration of guanabenz results in increased phosphorylation of eIF2α and modulates the key ERSR markers 6 hours post-SCI. (A) Schematic representation of guanabenz injections given at various time points-post SCI. (B,C) Western blots show that guanabenz significantly increases phosphorylated eIF2α levels at the injury epicenter of contused spinal cords. (D) Guanabenz leads to differential modulation in ERSR transcript levels as analyzed by qRT-PCR. Transcript levels are expressed as fold changes compared with respective levels in sham controls. Data are the mean ± SD [n = 3 (B,C); n = 4 (D), * p<0.05, **p<0.01].

To analyze the effects of guanabenz on the PERK-eIF2α pathway in the injured mouse spinal cord, animals were treated with intraperitoneal injection of 1 mg/kg of the drug immediately after injury (Fig 6A). A significant increase in levels of p-eIF2α was seen in guanabenz-treated animals at six hours post-injury (Fig 6B, C) indicating its effectiveness in enhancing this SCI-induced ERSR. Lack of any detectable p-eIF2α levels in the sham samples (Fig 6B) is consistent with our earlier study [18] and confirms the specific activation of ERSR post-SCI at the injury epicenter. Interestingly, while guanabenz significantly attenuated the levels of GADD34 and XBP1 at 6 hours post-injury, there was no significant difference in the ATF4 and CHOP transcript levels between vehicle- and guanabenz-treated animals (Fig 6D). These data demonstrated that guanabenz penetrated the spinal cord tissue *in vivo*, enhanced the PERK-eIF2α signaling and differentially modulated the key ERSR effectors after SCI.

### Guanabenz fails to improve functional recovery after SCI

**Figure 7:**
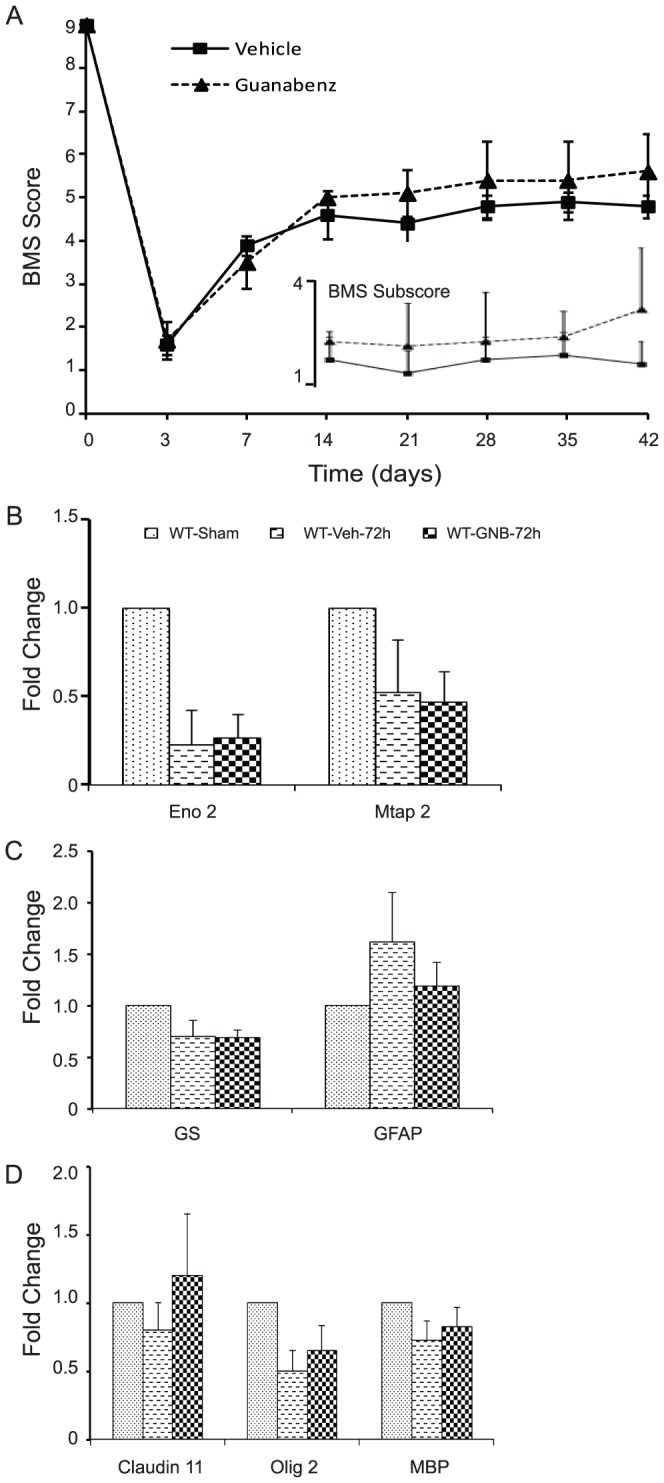
Administration of guanabenz does not enhance hindlimb locomotor function after SCI. (A) Open field BMS locomotor analyses performed weekly did not reveal any significant differences between vehicle- and guanabenz-treated animals. Analysis of BMS subscore also did not show any differences in stepping characteristics between the two groups (inset). Guanabenz treatment does not result in significant differences in the neuron- (B), astrocyte-(C) and oligodendrocyte-specific (D) transcript levels (as indicated) compared to vehicle-treated mice at 72 hours post-SCI. Data (A,B) are the mean ± SD (n = 6).

We next examined whether administration of guanabenz that resulted in differential modulation of the ERSR could enhance functional recovery after SCI. Analysis of BMS scores comparing vehicle- and guanabenz-treated animals over a period of six weeks post-injury revealed no significant differences between the two groups. The average BMS score of guanabenz-treated animals was 3.5±0.61 at week 1 which increased to 5.6±0.89 at week 6. Similarly, the average BMS score of vehicle-treated animals was 3.9±0.22 at week 1 which increased modestly to 4.8±0.27 (Fig 7A) at week 6. Analysis of detailed stepping characteristics using BMS subscore also failed to show any significant improvement in locomotion in animals treated with guanabenz (Fig 7A,inset). Furthermore, guanabenz treatment did not change the levels of neuron- (Fig 7B; NSE and Map2a,b), astrocyte- (Fig 7C; glutamine synthetase and GFAP) and oligodendrocyte-specific (Fig 7D; Claudin 11, Olig2, MBP) transcripts compared to vehicle-treated mice. These data are in contrast to our previous study where a significant increase in neuron- and oligodendrocyte-specific transcripts were observed with salubrinal treatment [22]. Thus, despite being pharmacologically active *in vivo*, guanabenz does not enhance functional recovery six weeks post-SCI.

## Discussion

One of the major acute pathophysiological outcomes after SCI is oligodendrocyte loss both in humans [34], [35] and rodents [36]. As white matter sparing is critical to functional recovery after thoracic contusive SCI [37], early pharmacological interventions that could reduce such oligodendrocyte loss might have important functional implications.

Recent studies established that the ERSR is induced in neurons, oligodendrocytes and astrocytes and plays a crucial role in the pathogenesis after SCI [17]–[20]. The ERSR-associated pro-apoptotic transcriptional regulator, CHOP was specifically upregulated in neurons and oligodendrocytes, but not in astrocytes of contused mouse spinal cords [18]. The sensitivity of oligodendrocytes to ER-stress mediated apoptosis is likely due to their highly developed ER serving the need to produce vast amounts of lipids and proteins for myelin synthesis [38], [39], [40]. SCI-associated dysregulation of intracellular Ca^2+^ homeostasis is one of the triggers of oligodendrocyte ER stress [41]. Our study using CHOP^-/-^ mice demonstrated a complete attenuation of the ERSR, increase in transcript levels of oligodendrocyte-specific MBP and claudin 11 at 72 hours and 6 weeks post-SCI, and a decrease in Olig2+ cells colocalized with cleaved caspase 3 at the injury epicenter and penumbra region [18]. Collectively, these data indicated that CHOP serves as an ERSR specific pro-apoptotic transcription factor in the context of SCI and oligodendrocytes are very sensitive to SCI-induced ER stress [18]. Pharmacological intervention into the PERK/CHOP arm of the ERSR with salubrinal, an inhibitor of the PP1 complex, similarly demonstrated recovery in hindlimb locomotor function [20] suggesting that restoration of ER homeostasis after SCI could be a therapeutically viable approach.

**Figure 8:**
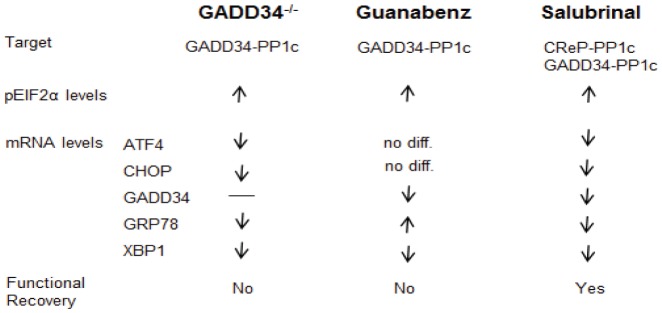
Schematic diagram showing the transcriptional and translational comparison and differences between the GADD34^-/-^, guanabenz- and salubrinal- treated mice.

Guanabenz (a FDA-approved drug) was recently identified in a chemical screen of drugs for phase II clinical trials to exhibit anti-prion activity [33]. Importantly, guanabenz selectively inhibited the stress-induced eIF2α holophosphatase by targeting its regulatory subunit (PPP1R15A/GADD34) without affecting the constitutive one (PPP1R15B/CReP) [22]. We previously showed that salubrinal, which targets both GADD34 and CReP, increased functional recovery after SCI [20]. We expected that guanabenz by selectively targeting the stress-induced GADD34, would similarly result in improved locomotor recovery post-SCI. However, guanabenz administration *in vivo* did not result in any reduction of locomotor deficits post-SCI. Comparing the effects of salubrinal [20] and guanabenz on SCI (Fig. 8), the simplest explanation would be that CReP is the more important phosphatase in the context of SCI. More likely, each of these drugs may inhibit non-overlapping PP1 complexes with different spectra of substrates. Therefore, their effects on ERSR and apoptosis may differ. This interpretation is supported by the data showing guanabenz treatment led to attenuation of GADD34 and XBP1 transcript levels only, having no significant effect on ATF4 and CHOP transcript levels. This is in contrast to salubrinal where a complete attenuation of the ERSR was observed [20]. Alternatively, the duration of phospho eIF2α levels that remain after SCI may be crucial. Salubrinal-treated mOPCs showed enhanced protection against tunicamycin and pEIF2α levels returning to basal levels by 16 hours [20] whereas guanabenz-treated mOPCs showed significant higher levels of pEIF2α at 24 hours post-treatment. This delay in translational recovery and subsequent return to cellular homoeostasis in guanabenz-treated mOPCs perhaps is detrimental to their survival. Consistent with this interpretation, it is well known that the resultant survival or cell death outcome of the ERSR is dependent on injury duration and involvement of different components of the ERSR [42]. Finally, the treatment regimen used for *in vivo* guanabenz administration was based on our previous study [20] and perhaps needs more standardization due to the varied stability and efficiency of the drug in context of SCI. Mice with genetic ablation of GADD34, a direct target of guanabenz, also failed to show any improvement in locomotor outcome. Lack of functional improvement coinciding with differential effects on ERSR suggests that the complex interplay of various components of the ERSR pathway is essential for cell survival. Since, GADD34 is an essential component of a negative-feedback loop operating under stress, blocking GADD34 either by pharmacologic or genetic means possibly results in compensatory changes to the ERSR activity that accounts for lack of functional improvement post-SCI.
